# Test validation, method comparison and reference range for the measurement of β-hydroxybutyrate in peripheral blood samples

**DOI:** 10.11613/BM.2020.010707

**Published:** 2020-02-15

**Authors:** Frank Bernhard Kraus, Marija Kocijancic, Alexander Kluttig, Beatrice Ludwig-Kraus

**Affiliations:** 1 Central Laboratory, University Hospital Halle, Halle (Saale), Germany; 2 Institute of Biology, Martin-Luther-University Halle-Wittenberg, Halle (Saale), Germany; 3 Institute of Medical Epidemiology, Biostatistics and Informatics, Martin-Luther- University Halle-Wittenberg, Halle (Saale), Germany

**Keywords:** β-hydroxybutyrate, ketoacidosis, method validation, reference range, urine ketones

## Abstract

**Introduction:**

The measurement of β-hydroxybutyrate (βOHB) concentrations is a corner stone of the diagnosis of diabetic ketoacidosis and other ketonic states. The aim of this study was to perform a validation of a peripheral blood βOHB assay (Randox) on a Roche cobas c502 analyser and to establish a βOHB reference range for the validated assay.

**Materials and methods:**

Precision, linearity and limit of detection and blank (LoD, LoB) were determined according to Clinical and Laboratory Standards Institute (CLSI) EP05-A3, EP 06-A and EP17-A2 guidelines, using commercial control material and residual patient sample pools. As method comparison, for 190 semi-quantitative measurements of urine ketones we determined the corresponding βOHB blood concentration. The reference range was based on the CLSI C28-A3 guideline, using 304 randomly selected serum samples from population based German National Cohort (GNC) study.

**Results:**

Coefficients of variation for the validated assay ranged from 1.5% for high concentrations (3.1 mmol/L) to 6.5% for low concentrations (0.1 mmol/L). Detection capacity was LoB = 0.011 mmol/L and LoD = 0.037 mmol/L. Linearity of the assay ranged from 0.10 to 3.95 mmol/L. The agreement between the semi-quantitative urine ketone test and the βOHB blood test was moderate (Kappa = 0.66). The obtained 95% serum reference range was estimated as 0.02 to 0.28 mmol/l βOHB.

**Conclusions:**

The Ranbut βOHB assay showed good precision and analytical performance. Our results confirm that βOHB measurement in peripheral blood is indeed a preferable alternative to the semi-quantitative measurement of urine ketones.

## Introduction

Ketosis is the physiological state in which insufficient supply of glucose leads to an increased β-oxidation of fatty acids and the production of the ketone bodies acetone, acetoacetate and β-hydroxybutyrate (βOHB) as an alternative primary energy source for the organism (*1*, *2*). Excessive production of ketone bodies leads to ketonemia and can ultimately result in ketoacidosis, a life threatening condition requiring immediate medical treatment. Ketoacidosis mostly occurs in diabetic individuals or under alcohol abuse in combination with fastening (*3*–*6*). More recently, with the increasing use of sodium glucose cotransporter 2 (SGLT2) inhibitors to treat diabetes type 2, the phenomenon of euglycemic diabetic ketoacidosis was recognized as a potential diagnostic blind spot and patient safety concern (*7*–*9*).

The laboratory quantification of ketone bodies is of crucial importance in the diagnosis and monitoring of ketoacidotic conditions. The measurement of ketone bodies in urine is commonly part of the routine urine laboratory analysis, since results can conveniently be obtained. Such urine dipstick tests are based on the reaction of nitroprusside with acetoacetate and are used in central laboratories as well as point of care testing (POCT). However, since the concentration of urine ketone bodies is not only influenced by the metabolic production of ketones, but also by factors like kidney function, hydration state or medication, the semi-quantitative urine ketone test is only a rough and time delayed estimate of the metabolic condition and ketonic state of a given individual (*10*, *11*). An alternative to the semi-quantitative urine ketone testing is the measurement of the concentration of the main ketone body β-hydroxybutyrate (βOHB) in plasma or serum. Obviously, the blood βOHB - concentration directly corresponds to the overall state of ketosis, without time delay or kidney function interference. While there have been quite a few validation studies concerning the measurement of capillary blood ketones *via* POCT analysers, studies concerning βOHB assays for peripheral blood seem to be lacking (*12*-*15*). The aim of study was to fill this gap and to perform a validation of a peripheral blood βOHB assay (Randox) on a Roche cobas c502 analyser and establish a reference range of βOHB concentrations for the validated assay.

## Materials and methods

### Study design

This study describes the validation of a quantitative βOHB assay on a Roche cobas c502 analyser, including the determination of precision and bias, testing linearity range of the assay as well as the estimation of the limit of blank (LoB), limit of detection (LoD) and limit of quantification (LoQ). To be able to gauge potential discrepancies between quantitative blood βOHB analysis and semi quantitative urine ketone testing, βOHB blood concentration ranges for corresponding semi-quantitative urine ketone categories were obtained and a method comparison was performed. Further, using randomly selected samples from the population based German National Cohort (GNC) from the study centre Halle (Saale), reference ranges for βOHB serum concentrations were established.

### Subjects

This comprehensive analytical validation study was carried out from October 2017 to February 2019 at the Central Laboratory of the University Hospital Halle (Saale). The first part of the study, the ßOHB test validation and method comparison study (both using residual anonymized inpatient and outpatient samples from routine diagnostics), was approved by the ethics committee of the Medical Faculty of the Martin-Luther-University Halle-Wittenberg (approval 2018-161). The patient samples used for the validation study consisted of 50 patient samples with ßOHB concentrations covering a wide measuring range, which were used to create plasma pools for the precision and linearity study. For the preparation of sample pools, sera were collected, mixed and frozen at − 20°C for 48 h, thawed for 1 day and afterwards filtered once using filter paper. For the method comparison study, urine and plasma sample pairs were randomly selected from 190 patients. The inclusion criterion was that both samples were taken at the same time. The general exclusion criterion for plasma samples was the presence of haemolysis, since haemolysis generally represents one of the most common source of interference in clinical chemistry, and so far there is no study available to our knowledge showing that haemolysis is not interfering with the measuring of ßOHB.

The second part of the study, the establishment of a reference range for peripheral blood ßOHB concentrations, used 304 outpatient samples from the German National Cohort (GNC) from the study centre Halle (Saale) (*16*). The GNC is a population based cohort study that aims to investigate the causes for the development of major chronic diseases, *i.e.* cardiovascular diseases, cancer or diabetes. Across Germany, a random sample of the general population (planned number of participants 200,000) is examined in 18 local study centres. The serum samples consisted of 155 men and 149 women aged 20-69 years with a median age of 50 years for both sexes. Since the GNC generally represents a population sample tilted towards more healthy individuals, no criteria according to patient diagnosis for blood sampling were included. The usage of GNC samples was approved by the GNC steering committee (approval NAKO-327). As an addition to the GNC samples used in the second part of the study 100 randomly selected serum and plasma samples pairs from routine diagnostics of the Central Laboratory were used to compare ßOHB concentrations in serum and plasma, because both sample types are used in our routine practice.

In accordance with Borovecki *et al*., signed informed consent for the usage of patient samples was deemed unnecessary, since throughout this study only residual samples were used and all patients’ data were anonymized (*17*). For GNC samples, participants signed informed consent.

### Blood sampling

All venous blood samples were collected in non-fasting state in standard plasma or serum tubes (S-Monovette Serum-Gel/S-Monovette Li-Heparin-Gel, 4.9 mL, Sarstedt, Nümbrecht, Germany) and centrifuged at 3000xg for 10 min at 18°C within a cobas 8100 pre-analytic unit. Urine samples were collected in standard urine tubes (Urin-Monovette, 8.5 mL, Sarstedt, Nümbrecht, Germany) and centrifuged at 800xg for 5 min using Rotina 35R centrifuge (Hettich, Tuttlingen, Germany).

### Validation of the Ranbut βOHB assay on a Roche cobas c502 analyser

The Ranbut βOHB assay (Randox Laboratories Limited, County Antrim, United Kingdom) is a kinetic enzymatic assay for the quantification of βOHB in serum or plasma samples. The method is based on the oxidation of βOHB to acetoacetate *via* the enzyme 3-hydroxybutyrate dehydrogenase and the simultaneous reduction of the co-enzyme NAD^+^ to NADH. The concurrent changes of the test solution’s absorption rate are directly proportional to the concentration of βOHB and can be photometrically measured at a wavelength of 340 nm. The assay is calibrated with a two-point calibration (Randox Calibration Serum Level 3, Randox Laboratories Limited, County Antrim, United Kingdom) according to the specifications and recommendations of the supplier and was implemented and measured on a Roche cobas c502 modular analyser (Roche Diagnostics, Rotkreuz, Switzerland). The cobas c502 module in the University Hospital Halle is part of a fully automated Roche cobas 8000 platform with a cobas 8100 pre-analytic unit and three cobas 8000 units. The Roche cobas 8000 platform and all its components are operated according to the manufacturer’s instructions and manuals, with routine maintenance and quality control procedures.

Imprecision and bias of the assay were determined according to the Clinical and Laboratory Standards Institute (CLSI) EP05-A3 guideline following a 2 x 2 x 20 experimental design (*18*). To calculate the precision of the assay, we used five test samples in total. These included two commercial control sera (Randox Assayed Multisera Level 2 and Level 3, Randox Laboratories Limited, United Kingdom) with specific βOHB target values (1.64 and 3.1 mmol/L), and three plasma based sample pools at low, medium and high βOHB concentrations. The three plasma pools were produced from residual, de-identified patient specimen from routine diagnostics covering a wide measuring range. The two control samples, with their defined target values, were also used for the estimation of the bias of the assay. The LoB, the LoD as well as the limit of quantification were determined following the CLSI EP17-A2 guideline using 120 blank and 120 low concentration measurements (*19*). Saline solution (0.9%) was used for the creation of 10 blank samples, with each of them being measured over three days with four measurements each day. Ten residual, de-identified patient plasma samples were diluted with saline solution to obtain βOHB concentration samples close to the LoB of the assay, and were measured four times each on three consecutive days to estimate the LoD.

Linearity was evaluated following the CLSI EP06-A guideline (*20*). Two patient sample pools, one with a concentration of 0.01 mmol/L and a pool of 3.95 mmol/L were used to create a dilution series of 10 concentration levels with four measurements at each concentration. The maximum obtainable βOHB concentration for the sample pool was ultimately determined by the availability (and general scarcity) of high concentration patient samples during linearity testing. The goal for the allowable error (18.7%) of the linearity testing was calculated as desirable bias based on the biological variation of βOHB (*21*, *22*).

### Stability of βOHB in plasma samples

To test the stability of βOHB in plasma samples under standard refrigerator temperatures, 10 plasma samples (residual, de-identified patient specimen) were measured once *per* day for a one-week period, representing the maximum refrigerator storage time planned for βOHB samples in the Central Laboratory of the University Hospital Halle (Saale). Baseline readings were taken from each sample within 1 hour of collection. After the baseline readings samples were stored in cobas p501 post-analytical unit at a range of 4 to 8°C, and analysed after 1, 2, 3, 4, 5 and 6 days of storage. Since the cobas p501 unit is connected to the cobas 8100 automated workflow, all samples are closed using a flexible archiving cap and stored immediately into refrigerator after measurement.

### Method comparison - urine ketones vs. βOHB plasma concentration

Urine ketone bodies were measured on an automated iChemVELOCITY analyser (Beckman Coulter Life Sciences, Krefeld, Germany) with urine test strips. The test for ketone bodies in urine is based on the coupling of methylketone with glycine and sodium nitroprusside in alkaline buffer. Proportional to the ketone concentration the reaction produces a violet colour, which is measured with reflectance photometry. The results of the urine ketone test are given as five, semi-quantitatively categories (negative, +, ++, +++, ++++). For 190 patient urine samples from routine diagnostics, where simultaneously plasma samples had been taken, the βOHB plasma concentration was measured, on the same day as the semi-quantitatively urine ketone test, to obtain a corresponding βOHB plasma concentration for each of the urine samples.

### βOHB reference range

To obtain a βOHB reference range for the Randox βOHB assay, βOHB concentrations of randomly selected 304 serum samples obtained from participants of the German National Cohort (GNC) study from the study centre Halle (Saale), were measured and subsequently analysed following the CLSI C28-A3 guideline (*23*). On days (weekends and holidays) when analysis could not be performed, samples were stored in the refrigerator (4-8°C) until analysis, thus having been stored for a maximum of three days before measurement. In addition, to be also able to estimate a βOHB reference range for plasma samples, a comparison of βOHB concentrations in 100 serum and plasma sample pairs (residual de-identified patient specimen from routine diagnostics) was performed.

### Statistical analysis

The statistical analysis of the validation of the Ranbut βOHB assay was carried out according to CLSI guidelines (*18*-*20*, *23*). The imprecision (as coefficient of variation) of the assay was calculated with an ANOVA and bias was estimated as relative and absolute deviation from the target values (CLSI EP05-A3). To calculate the statistical significance of the bias, we used the statistical approach described in the CLSI EP05 and EP15 guidelines, which estimate the 95% confidence interval of the bias (mean value *vs.* target value). Linearity was assessed by a polynomial regression analysis to first-, second- and third-order polynomials (CLSI EP 06-A). The LoB was calculated as: mean_(blank)_ + 1.645 SD_(blank),_ where mean_(blank)_ is the arithmetic mean of blank samples and SD_(blank)_ their corresponding standard deviation. The LoD was calculated as: LoB + 1.645 SD_(pooled low)_, where SD_(pooled low)_ is the pooled standard deviation of the diluted low concentration samples. The CVs of the low concentration samples were used to estimate the LoQ of the assay (CLSI EP17-A2). The stability of βOHB concentrations in plasma samples over a one-week period was tested with a purely statistical approach, using a Friedman test.

We used the interrater-reliability kappa statistic to quantify the agreement between the semi-quantitative urine ketone test and the measurement of βOHB concentrations in plasma (*24*, *25*). As an additional statistical approach to quantify the agreement between the two tests, we used a Kruskal–Wallis analysis of variance to test for differences between the urine ketone test categories (negative, +, ++, +++, ++++) concerning their associated plasma sample βOHB concentrations.

Based on the 304 serum samples obtained from participants of the GNC public health study, the reference range was calculated with a non-parametric percentile method as recommended by the CLSI C28-A3 guideline (*23*). We tested for significant differences in βOHB concentrations between men and women using a Mann Whitney test.

To test whether serum and plasma sample pairs (N = 100) differ significantly in their βOHB concentrations we used a Wilcoxon test for paired samples. The average difference between βOHB concentrations in serum and plasma sample pairs was then calculated as the mean relative difference. This mean relative difference was subsequently applied as a conversion factor to the individual serum βOHB concentrations of the GNC study population to obtain corresponding βOHB plasma concentration estimates. These plasma concentration estimates were then used to calculate a corresponding reference range for βOHB plasma, again with a non-parametric percentile method as described above (*23*).

All statistical analyses were carried out with the Microsoft Excel 2010 add-in Analyse-it (Method Validation edition; Analyse-it for Microsoft Excel 5.11) or the statistic software MedCalc (Version 18.11.3, MedCalc Software, Mariakerke, Belgium).

## Results

### Validation of the Ranbut βOHB assay on a Roche cobas c502 analyser

The precision of the Ranbut βOHB assay was good, with the total (within-laboratory) coefficient of variation (CV) ranging from 1.5% for high concentration samples (3.1 mmol/L), up to 6.8% for low concentration samples (0.13 mmol/L). The repeatability (within series) CV contributed the most to the overall variation of the assay. The obtained CVs of the control samples (QC1, QC2) were in line with those of the plasma pool samples (pool 1, 2 and 3). The bias estimate based on the control samples was significantly larger than zero for both QC1 and QC2 (P < 0.001 and P = 0.024 respectively), with - 0.02 and 0.01 mmol/L absolute bias from the target values (6.7% and 0.6% relative bias). However, taking into account the biological variation of βOHB concentrations, which is 18.7%, these biases were considered clinically not significant (*18*, *20*). A detailed account of the results for bias and precision is given in Table 1.

**Table 1:** Results for bias and precision testing of the Ranbut βOHB assay on a Roche cobas c502 analyser according to the CLSI EP05-A3 guideline

**Sample**	**Mean,** **mmol/L**	**Target value,** **mmol/L**	**Bias,** **mmol/L**	**Relative bias,** **%**	**Repeatability SD,** **mmol/L**	**Repeatability CV,** **%**	**Total SD** **mmol/L**	**Total CV** **%**
QC1	0.27	0.29	- 0.02	- 6.7	0.010	3.7	0.013	4.7
QC2	1.16	1.15	0.01	0.6	0.012	1.0	0.018	1.6
pool 1	0.13	-	-	-	0.007	5.0	0.009	6.8
pool 2	1.64	-	-	-	0.023	1.4	0.034	2.1
pool 3	3.10	-	-	-	0.045	1.4	0.048	1.5
QC1 and QC2 are control samples, while pool 1, 2 and 3 are plasma pool samples. Given for each sample are the mean of the βOHB measurements in mmol/l and for QC1 and QC2 the assigned target values (mmol/L) as well as the resulting absolute (mmol/L) and relative bias from the respective target value. For all samples the repeatability and total standard deviations (SD, mmol/L) and the repeatability and total coefficients of variation (CV) are given. QC – quality control.								

The LoB of the assay was estimated to be 0.011 mmol/L and the corresponding LoD 0.037 mmol/L. The CVs for the measurements of the 10 low concentration samples, which were used for the determination of the LoD, were high, ranging from 16.1% to 159.1% with the CVs increasing with decreasing βOHB concentrations. Based on the low concentration sample CVs, the estimated LoQ, below which the CV is expected to exceed 20% (CLSI EP17-A2), was at 0.04 mmol/L.

Overall, the tested dilution series (concentration range 0.005 mmol/L to 3.95 mmol/L) deviated significantly from linearity with a second order polynomial fitting statistically better than a linear fit at the 5% significance level. The goal of the allowable error for the linearity testing was calculated as 18.7%, based on the biological variation of βOHB (*21*, *22*). Comparing the allowable error goal to the estimated non-linearity at each dilution level, the assay however is linear between 0.103 and 3.95 mmol/L. The detailed results of the linearity testing are given in Table 2.

**Table 2:** Results of the linearity testing of the Ranbut βOHB assay on a Roche cobas 502 analyser according to the CLSI CLSI EP06-A guideline

**Dilution**	**Mean**	**SD**	**Linear fit**	**Non-linear fit**	**Non-linearity**	**95%CI**
**L**	0.005	0.006	0.033	0.002	- 95.1%	- 115.5 to - 74.8%
**0.99L + 0.01H**	0.055	0.006	0.073	0.045	- 37.5%	- 45.5 to - 29.5%
**0.97L + 0.03H**	0.103	0.005	0.152	0.133	- 12.8%	- 15.5 to - 10.1%
**0.95L + 0.05H**	0.228	0.005	0.232	0.220	- 5.2%	- 6.3 to - 4.1%
**0.9L + 0.1H**	0.433	0.009	0.431	0.435	1.1%	0.9 to 1.3%
**0.8L + 0.2H**	0.885	0.006	0.828	0.860	3.9%	3.0 to 4.7%
**0.6L + 0.4H**	1.663	0.015	1.624	1.684	3.7%	2.9 to 4.5%
**0.4L + 0.6H**	2.493	0.028	2.420	2.472	2.2%	1.7 to 2.6%
**0.2L + 0.8H**	3.205	0.037	3.215	3.225	0.3%	0.2 to 0.4%
**H**	3.95	0.036	4.011	3.942	- 1.7%	- 2.1 to - 1.3%
The mean, its standard deviation (SD), linear fit and non-linear fit (2nd order polynomial) are given in mmol/L. Non-linearity and its 95% confidence interval (95%CI) are given in percentage. L – low pool. H – high pool.						

### Stability of βOHB in plasma samples

The βOHB concentration of the used samples ranged from 0.01 mmol/L up to 0.73 mmol/L. As such, one sample had to be excluded from further analysis, since it proved to be below the LOD of the assay. When plasma samples were stored for one week at 4-8 °C and daily tested, there was no change in plasma βOHB concentration over time (Friedman test: F = 0.739; P = 0.621). The measured sample concentrations are given in Table 3.

**Table 3:** Stability of ßOHB over 7 days storage at 4 - 8°C

**Samples**	**T_0_**	**T_1_**	**T_2_**	**T_3_**	**T_4_**	**T_5_**	**T_6_**
**1**	0.06	0.06	0.06	0.04	0.05	0.05	0.05
**2**	0.06	0.06	0.05	0.04	0.06	0.05	0.06
**3**	0.06	0.06	0.04	0.08	0.05	0.06	0.05
**4**	0.11	0.11	0.08	0.07	0.07	0.08	0.09
**5**	0.17	0.17	0.19	0.18	0.16	0.18	0.18
**6**	0.17	0.17	0.17	0.17	0.16	0.17	0.17
**7**	0.24	0,24	0.23	0.24	0.26	0.26	0.25
**8**	0.38	0.39	0.38	0.38	0.39	0.39	0.39
**9**	0.72	0.73	0.73	0.73	0.73	0.72	0.73
T_0_ = ßOHB concentration (mmol/L) baseline value. T_1_ – T_6_ = ßOHB concentration (mmol/L) at day 1 to 6 after baseline measurement and storage at 4-8 °C. A Friedman test did not reveal any difference between the measurements over the storage period (F = 0.739, P = 0.621). βOHB - β-hydroxybutirate.							

### Method comparison - urine ketones vs. βOHB plasma concentration

The interrater agreement analysis resulted in a Kappa of 0.66 (95% CI: 0.55 to 0.77), and thus the agreement between the urine ketone test and the βOHB plasma test can be considered moderate.

The Kruskal Wallis analysis showed, that while the βOHB plasma concentration ranges associated with the five semi-quantitative urine ketone categories, overall differed significantly from each other (Kruskal Wallis test: P < 0.001), they showed considerable overlap as presented in Table 4. A Conover *post-hoc* analysis further revealed that all semi quantitative urine ketone categories were statistically different from each other concerning their associated βOHB plasma concentrations at the P < 0.05 significance level. However, when applying a stricter significance threshold of P < 0.01 in the Conover *post-hoc* analysis the ++ and +++ urine ketone categories did not differ from each other anymore.

**Table 4:** Comparison of median βOHB plasma concentrations among five semi-quantitative urine ketone categories

**Category**	**N**	**Median (IQR)**	**Range**
negative	46	0.06 (0.02–0.18)	0.00–0.69
+	40	0.53 (0.13–0.75)	0.00–2.42
++	40	0.95 (0.60–1.50)	0.22–3.21
+++	40	1.49 (0.70–3.06)	0.05–6.82
++++	24	2.77 (2.04–5.60)	0.53–10.53
diab. ketoacid.	11	5.72 (3.85–7.59)	3.34–10.53
Shown are βOHB concentrations (mmol/L) of 190 plasma samples distributed over their associated five semi-quantitative urine ketone-bodies categories from corresponding urine samples and, as a sub set, for patients with diagnosed diabetic ketoacidosis. Given is the median and interquartile range (IQR), as well as the concentration range for each category in mmol/L. βOHB - β-hydroxybutirate.			

As expected, individuals with a diabetic ketoacidosis in the method comparison (N = 11) had very high βOHB concentrations in plasma (median = 5.72 mmol/L, interquartile range (IQR) = 3.85 – 7.59 mmol/L) and were categorized as +++ (N = 5) and ++++ (N = 6) categories by the semi-quantitative urine ketone body test.

### βOHB reference range

Based on the 304 samples from the GNC, the serum βOHB 95% reference range for women and men was estimated as ranging from 0.02 (90%CI: 0.01 – 0.02) mmol/L to 0.28 (90%CI: 0.25 – 0.50) mmol/L (CLSI C28-A3, non-parametric percentile method). Overall, women and men did not differ in their βOHB concentrations (Mann-Whitney test; P = 0.195; women N = 149, median women = 0.05 mmol/L, IQR women = 0.03 to 0.07 mmol/L; men N = 155, median men = 0.05 mmol/L, IQR men = 0.04 to 0.09 mmol/L).

The comparison of βOHB concentrations in serum - plasma sample pairs (N = 100) showed that serum samples yield a mean 0.02 mmol/L (24.1%) higher βOHB concentration than the corresponding plasma samples. Applying this conversion factor, the reference range for plasma samples was estimated as 0.01 (90%CI: 0.00 – 0.01) to 0.25 mmol/L (90%CI: 0.22 – 0.47) βOHB.

## Discussion

The validation of the βOHB assay (Ranbut) on a Roche cobas c502 analyser described in this study yielded overall good performance characteristics. The assay is precise, with the CV decreasing with increasing βOHB concentrations (6.8% to 1.5%; Table 1). Similar CVs (3.8% to 5.3%) are claimed by the manufacturer for the Ranbut βOHB assay, albeit on a RX Monza analyser (Randox), with lower sample sizes (N = 20) and at only two concentration levels (0.32 and 1.17 mmol/L). In addition, the estimated bias was generally low, with the higher value again found in the low concentration range (-6.7% and 0.8% bias, respectively). As such, the assay is highly reliable in the clinical relevant, ketonamic concentration ranges, while having markedly higher, but still acceptable, values for imprecision and bias in the clinically not relevant concentration range. With 0.01 mmol/L βOHB as LoB and 0.04 mmol/L as LoD the assay has a very high detection capacity and, based on these results, the estimated LoQ (below which the CV is expected to exceed 20%) was at 0.04 mmol/L. However, the assay’s limiting factor for the measuring range is the linear range, rather than the very low LoD and LoQ. Below βOHB concentrations of 0.1 mmol/L the predefined acceptable error goals could no longer be met, leading to a linear measuring range of 0.1 to 3.95 mmol/L βOHB. This is in accordance with the assay’s manufacturer linearity claim of 0.1 and 3.2 mmol/L, where samples with βOHB concentrations above the linear range can be diluted 1:3, which would lead to a final measuring range of up to 9.6 mmol/L for the application on a Roche cobas c502 analyser. The analyte stability evaluation carried out in this study, indicates that βOHB plasma samples can reliably be stored at 4 - 8°C for at least 6 days, without any significant decrease in βOHB concentrations. For the purposes and needs of most laboratories, this period should be more than sufficient, given that βOHB measuring is anyway often an emergency parameter and would then require immediate measurement. Similar findings are provided in a study by Carragher *et al.*, where ßOHB is claimed to be stable in plasma for up to 7 days at room temperature, 14 days at 4°C and 6 months at –20 °C (*26*).

The comparison of the Ranbut βOHB assay with the semi-quantitative urine ketone assay *via* the interrater-agreement kappa statistic, showed weak to moderate agreement when taking into account the confidence intervals of the Kappa estimate (Kappa = 0.66; 95%CI: 0.55 to 0.77) between the two tests (*25*). However, the Kruskall-Wallis test, which uses more information from the obtained data set than the Kappa statistic, showed that the semi-quantitative categories significantly differ in their associated plasma βOHB concentrations. Nevertheless, all five semi-quantitative categories show considerable overlap of their corresponding plasma βOHB concentrations, rendering a clear allocation of a given individual urine sample to a clearly defined βOHB blood concentration category impossible (Table 4). This only moderate agreement between the two test systems is however not surprising given the time delay between the occurrence of ketones in blood and urine and the influence of factors unrelated to ketone production like hydration state and kidney function on the urine ketone concentration measurement (*10*, *11*).

Based on the 304 samples of the GNC study, the calculated serum 95% reference range for βOHB was 0.02 to 0.27 mmol/L. Applying the conversion factor obtained from the serum-plasma βOHB concentration comparison, the normal 95% range for plasma samples is expected to be 0.01 to 0.24 mmol/L βOHB. These reference ranges are similar to the manufacturer claim of 0.03-0.3 mmol/L as “normal value” for plasma samples, even though no specifications on the sampled population or the statistical approach are provided. Similar to our reference range, several authors define βOHB concentrations above 0.3 mmol/l as already significantly elevated, even though these estimates are based on capillary βOHB concentrations (*27*, *28*).

Applying our reference range for plasma samples (0.01-0.24 mmol/L) to the comparison of the semi quantitative urine ketone assay with the βOHB concentrations in corresponding plasma samples, the semi quantitative urine ketone assay included 17% individuals above the reference range in the negative category. Similar to this, the single positive (+) urine ketone category included 35% individuals with βOHB plasma concentrations within the reference range. These mismatches might be interpreted as false negative and false positive cases in the semi quantitative urine ketone assay, even though one might argue that it is not clear from which concentration on elevated βOHB plasma concentrations are truly pathological. One often cited “cut-off” for βOHB concentrations in ketoacidotic conditions is 3.0 mmol/L, which leaves a considerable grey area of elevated but not ketoacidotic βOHB concentrations (*27*, *29*). Interestingly patients with a defined diabetic ketoacidosis had βOHB concentrations ranging from 2.9 up to 10.5 mmol/L, which is fitting well to the aforementioned cut-off of 3.0 mmol/L.

Overall, the assay proved to be analytically superior to the semi-quantitative urine ketone testing, providing the clinician with a much more precise and reliable detection and monitoring tool for patients with ketosis. Given the increasing use of SGLT2 inhibitors in diabetic patients, the measurement of blood βOHB will become even more important, since urinary ketone monitoring is not recommended in such patients (*7*, *30*).

So, while for diabetic patients measuring βOHB levels at home might be best realized with on-site POCT analysers, the here described βOHB assay on a large clinical chemistry analyser can be valuable asset for central laboratories especially for hospitals with emergency wards and intensive care units.
